# Supplementary material for: Conditional chemoconnectomics (cCCTomics) as a strategy for efficient and conditional targeting of chemical transmission
Source: eLife. 2024 Apr 30;12:RP91927. doi: 10.7554/eLife.91927 (PMC11060718; doi:10.7554/eLife.91927)
Supplement: Figure 3—source data 3. [file elife-91927-fig3-data3.zip › Figure 3 source data3 part 2.zip!/Figure 3-Source data 3-part2/1062-13-CRISPResso_Report_jt28fhr9.zip!/CRISPResso_on_1062_15/9.gcaggaaatgagctacctac.Alleles_frequency_table_around_sgRNA_gcaggaaatgagctacctac.pdf]

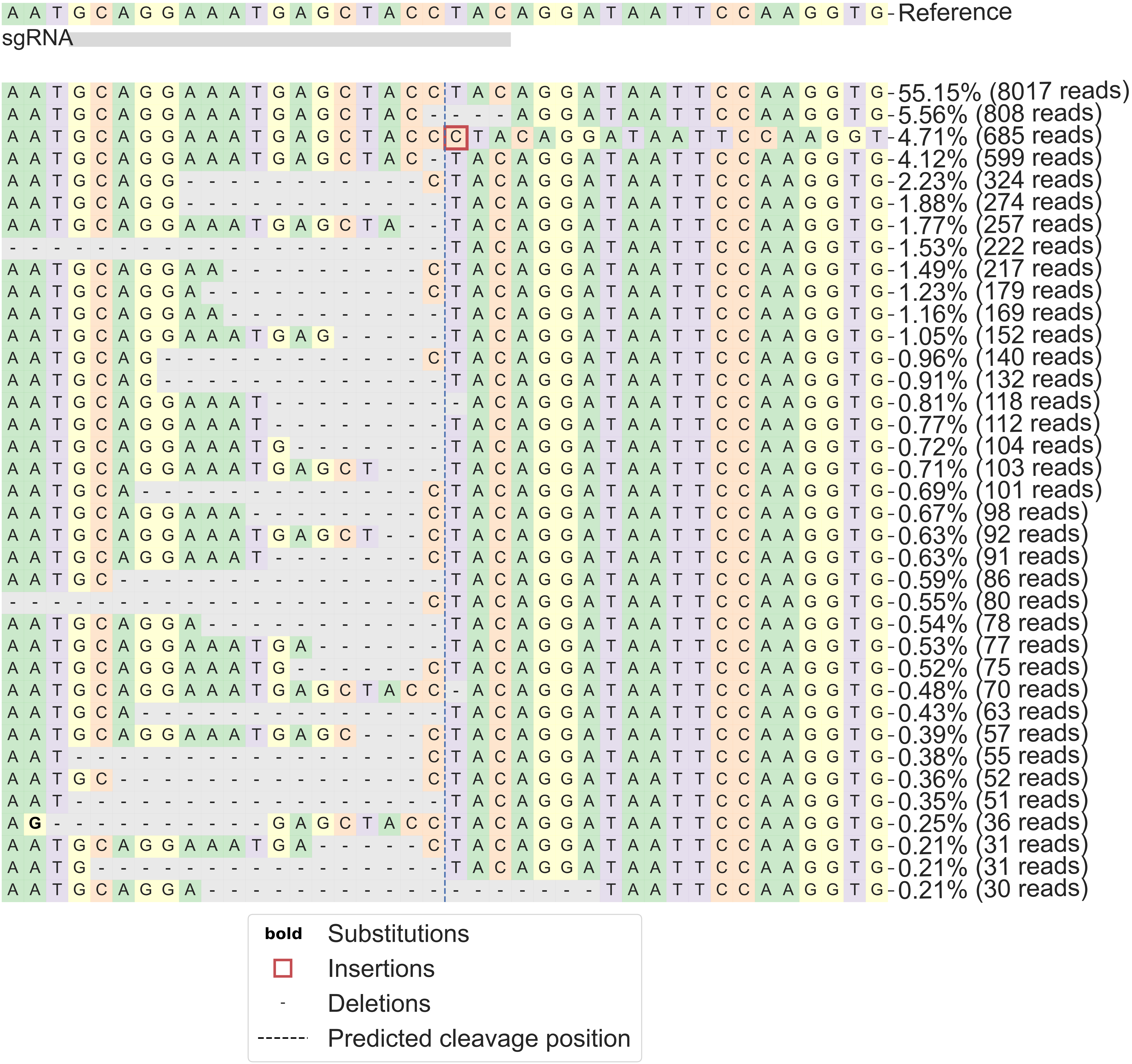
A A T G C A G G A A A T G A G C T A C C T A C A G G A T A A T T C C A A G G T G Reference
sgRNA
55.15% (8017 reads)
5.56% (808 reads)
4.71% (685 reads)
4.12% (599 reads)
2.23% (324 reads)
1.88% (274 reads)
1.77% (257 reads)
1.53% (222 reads)
1.49% (217 reads)
1.23% (179 reads)
1.16% (169 reads)
1.05% (152 reads)
0.96% (140 reads)
0.91% (132 reads)
0.81% (118 reads)
0.77% (112 reads)
0.72% (104 reads)
0.71% (103 reads)
0.69% (101 reads)
0.67% (98 reads)
0.63% (92 reads)
0.63% (91 reads)
0.59% (86 reads)
0.55% (80 reads)
0.54% (78 reads)
0.53% (77 reads)
0.52% (75 reads)
0.48% (70 reads)
0.43% (63 reads)
0.39% (57 reads)
0.38% (55 reads)
0.36% (52 reads)
0.35% (51 reads)
0.25% (36 reads)
0.21% (31 reads)
0.21% (31 reads)
0.21% (30 reads)
bold Substitutions
Insertions
Deletions
Predicted cleavage position
